# Ethnicity and Language Proficiency Differences in the Provision of and Intention to Use Prenatal Screening for Down’s Syndrome and Congenital Anomalies. A Prospective, Non-selected, Register-Based Study in the Netherlands

**DOI:** 10.1007/s10995-017-2364-2

**Published:** 2017-09-07

**Authors:** Ingrid A. Peters, Kirsten M. Heetkamp, Nicolette T. C. Ursem, Eric A. P. Steegers, Semiha Denktaş, Maarten F. C. M. Knapen

**Affiliations:** 1 Division of Obstetrics and Prenatal Medicine, Department of Obstetrics and Gynaecology, Erasmus University Medical Center, Wytemaweg 80, Na-1515, 3015 GE, 3000 CA Rotterdam, The Netherlands; 2 Foundation Prenatal Screening Southwest Region of the Netherlands, Wytemaweg 80, Na-1509, 3015 GE Rotterdam, The Netherlands; 3 Department Social and Behavioural Sciences, EUC/Erasmus University Rotterdam, Nieuwemarkt 1a, 3011 HP Rotterdam, The Netherlands; 4 Research Centre Innovations in Care, Rotterdam University of Applied Sciences, Rochussenstraat 198, 3015 EK Rotterdam, The Netherlands

**Keywords:** Prenatal screening, Ethnicity, Language proficiency, Inequalities, Counseling

## Abstract

*Objective* We aimed to conduct an analysis of the associations between the information provision procedure of prenatal screening for Down’s syndrome and congenital anomalies and the intention to participate in prenatal screening (PS) of ethnicity groups and Dutch language proficiency groups. *Design* Using a prospective web-based registration form, we asked counselors (midwives, general practitioners, nurses and gynecologists) to report whether and how they offered information about PS to pregnant women. *Duration* The study was conducted from 2008 to 2010. *Participants* We collected data on the characteristics of the women who received an information offer about PS from counselors. *Measurements* Measures included socio-demographic and language proficiency level (LPL) characteristics, key elements of the provision procedure of PS, and intentional participation in PS. *Findings* The dataset represents 37% of the total population in the study area. Women with a non-native Dutch background and/or insufficient Dutch LPL received fewer information offers about PS, faced a reduced chance of receiving counseling, and showed lower intentional participation rates for PS. *Key Conclusions* Women with a non-native Dutch background and/or with an insufficient LPL are underserved in the Dutch PS program. These findings present evidence indicating that the fundamental principle of the Dutch Population Screening Act, namely, equal access to PS for all pregnant women, is not being realized. *Implications for Practice* Therefore, the study findings are important for national and international healthcare, policy makers and governmental professionals to allow ethnic and LPL-related differences in the provision and intentional uptake of PS.

**Electronic supplementary material:**

The online version of this article (doi:10.1007/s10995-017-2364-2) contains supplementary material, which is available to authorized users.

## Significance

*What is already known on this topic?* Several studies have shown that there are disparities in Dutch prenatal screening (PS). Ethnic minority groups are less likely to make an informed decision and participate in antenatal care due to the existence of possible language, cultural, and religious barriers; health illiteracy; being relatively underserved in terms of health services; and the absence of culturally sensitive information within the health services. 

*What does this study add?* This study shows that pregnant women with a non-native Dutch background and/or with insufficient Dutch language proficiency (LPL) are underserved more often within the Dutch PS program. These women are less likely to receive an information offer about PS and to receive counseling. The Population Screening Act ‘prenatal screening’ calls for equal access to the program. Therefore, these study findings are important for healthcare practitioners, policy makers and governmental professionals.

## Introduction

**Fig. 1:**
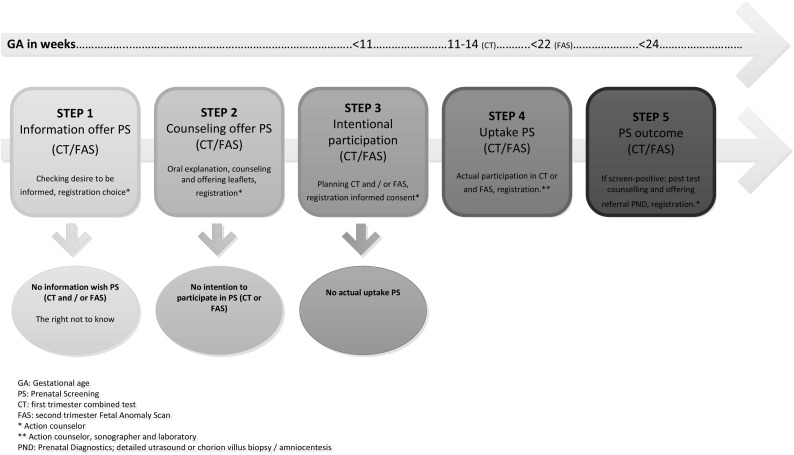
Dutch prenatal screening on Down’s syndrome and congenital anomalies

Since 2007, a nationwide prenatal screening program was introduced in the Netherlands. The program is supported by a legislative framework (the Population Screening Act), providing standards for regional and nationwide coordination and quality assessment of prenatal screening (Health Council of the Netherlands 2007). The Screening Act calls for equal access to the program for all pregnant women. According to this act, pregnant women who indicate that they require information should be counseled on the first trimester combined test (CT) for Down’s syndrome and the second trimester fetal anomaly scan (FAS) for congenital anomalies (Health Council of the Netherlands 2007; Wald and Hackshaw 1997; Bricker et al. 2000). The aim of offering counseling is to foster informed decision making (Marteau et al. 2001). For an overview of the Dutch prenatal screening program, see Fig. 1.

International differences in screening policies (for example, CT as an extra option in the Netherlands or as part of routine care in other countries) have affected the provision procedures and, therefore, the informed decision making and the uptake of CT among women (Vassy et al. 2014; Crombag et al. 2013, 2014; Dormandy et al. 2002). In the Netherlands, advanced maternal age-related risk perception, the financial threshold for younger women for the CT (until 2014) and the relatively positive attitude towards Down’s syndrome are likely to have a negative effect on CT uptake (Crombag et al. 2015; Bakker et al. 2012). The FAS is considered standard care by non-Dutch pregnant women, which possibly influences the current participation rate, which is comparable with that of the native Dutch population (89 vs. 90%, respectively, during the Deliver study 2009–2011) (Gitsels-van der Wal et al. 2014a, b, c; Nivel 2015).

The legislative framework of the Dutch prenatal screening program prescribes that all pregnant women, regardless of their ethnic background or Dutch language proficiency, must have equal access to the prenatal screening program. Nevertheless, several studies have shown that there are disparities in Dutch prenatal screening. Ethnic minority groups are less likely to make an informed decision and participate in antenatal care due to the existence of possible language, cultural, and religious barriers; health illiteracy; being relatively underserved in terms of health services; and the absence of culturally sensitive information within the health services (Crombag et al. 2015; Gitsels-van der Wal et al. 2014a, b, c; Fransen et al. 2010a, 2009; Woloshin et al. 1997; Andrulis 1998; Dormandy et al. 2005; National Institute for Public Health & Environment 2011). Multiple studies endorse the association between lower intentional and actual participation in the CT and partly endorse the association between ethnicity and language proficiency level (LPL); however, research on these outcomes for the FAS is lacking (Bakker et al. 2012; Fransen et al. 2010b; Dormandy et al. 2005). The aim of this study is to analyze the association between the individual offer of prenatal screening with the CT and FAS and ethnic and language proficiency groups within a population of pregnant women and their intention to participate in prenatal screening.

## Methods

### Data Collection

The study was performed in the southwestern region of the Netherlands, the largest prenatal screening region in the country. In comparison to other Dutch regions, the southwestern region is characterized by a larger urban and suburban area and a relatively high percentage (24%) of non-western immigrants (Statistics Netherlands 2013).In this prospective study, we used data obtained through a web-based registration form, in which the regional center for prenatal screening asked counselors (midwives, nurses and gynecologists) to report for each pregnant woman in their practice whether and how they were offered information about prenatal screening during the period between June 2008 and December 2010. This registration form was primarily intended for quality control of the contracted healthcare professionals by the regional center in the southwestern part of the Netherlands. The legal use of anonymous data from the registration form was based on the ‘implied consent’ of pregnant women who had received an information offer of prenatal screening and/or who participated in the program. From April to December 2010, the regional center included four extra variables in the registration form, including voluntary registration of the ethnicity of the pregnant women. Healthcare professionals eligible for this study practiced in one of the hospitals or community midwifery practices contracted by the regional center for PS in the southwestern region of the Netherlands. Fifty-two contracted organizations contributed to registration, which corresponds to 50% of the total number of contracted counseling organizations, including five general practitioner practices, two secondary hospitals and 45 midwifery practices.

### Measurements

For more detailed information about the registration of women’s socio-demographic and pregnancy characteristics, see Online Appendix 1. The registration form was based on the criteria for comprehensive counseling. The three key elements of counseling were registered. These key elements included the following: (1) pregnant women’s ‘wish to be informed’ and, if desired, (2) the actual provision of information about the CT and FAS (‘counseling’) and (3) the obtaining of ‘informed consent’. Because reliable uptake numbers for the CT and FAS were lacking during the study period, the intention to participate in the CT and FAS (planning uptake) was registered. For this study, we used the data of pregnant women who primarily received an information offer about prenatal screening. The dataset contained information on 37% of the total population of pregnant women in the southwestern region of the Netherlands (Table 1) (Statistics Netherlands 2013).

**Table 1:** Background characteristics of pregnant women who were offered counseling about prenatal screening

	Web application registrations June 2008–December 2010 (n = 30,549)			Exact numbers (n = 60,038)^a^	
	N	%	Median (range)	N	%
Age (years)	30,095		30 (11–50)		
≤19	550	2			
20–29	13,394	44			
30–39	15,375	51			
40–50	776	3			
Age category					
<36	25,911	86^b^		47,791	80
≥36	4184	14		12,247	20
Ethnic origin^e^	6083			17,990^c^	
Dutch	4018	66		11,133	62
Surinamese	187	3		817	5
Antillean	150	2		484	3
Cape Verdean	64	1		187	1
Turkish	307	5		974	5
Moroccan	470	8		1116	6
Other	887	15		3279	18
Generation^e^	6083			17,990^c^	
First-generation immigrants	1880	31		4430	25
Second-generation immigrants	185	3^d^		2427	13
Native Dutch	4018	66		11,133	62
Parity	30,229		1 (0–14)		
Nulliparity	15,065	50			
Multiparity 1–3	14,635	48			
Multiparity 4–14	529	2			
Gravidity	30,182		2 (0–20)		
1–2 pregnancies	21,707	72			
>2 pregnancies	8475	28			
Gestational age (first booking visit)	29,007		9 (5–41)		
0–11 weeks (on time)	22,894	79			
12–41 weeks (too late)	6113	21			
Dutch language proficiency level	28,043				
Absent	583	2			
Limited	1259	5			
Fluent	26,201	93			
Urbanity	30,549				
Not or less urban	6397	21			
Moderately urban	4841	16			
Highly urban	19,311	63			

^a^Exact numbers of mothers that gave birth to a living child in the study area from June 2008 to December 2010 [Data request, CBS Statistics Netherlands 2013]

^b^Higher representation of pregnant women <36 years old due to more inclusion by midwifery practices (low risk population)

^c^Exact ethnicity distribution for the study area. To make the study outcomes comparable with the denominator data, only 9 months of the year 2010 were explored

^d^Under-reporting second generation caused by the strict definition of second generation (country of birth of the pregnant women and both partners most be known)

^e^Ethnicity and immigrant generation variables only registered from April 2010 to December 2010

Information on the provision of information about prenatal screening (counseling) was obtained using several multiple-choice questions: “Is the willingness to receive information on the CT/FAS verified?” [yes/no/not applicable], “Have the women already been counseled for the CT/FAS?” [Yes, already counseled or will receive counseling in special counseling consultation/Yes, but not in a special counseling consultation/No or Not applicable] and “How is information on the CT/FAS actually provided?” [The multiple-choice question allows respondents to select more than one answer. Selection of options: ‘leaflet’, ‘counseling, verbal explanation’, ‘website’ and ‘other’]. The results for intentional participation in PS were obtained with two multiple-choice questions: (1) “Does the pregnant woman wish to participate in CT?” and (2) “Does the pregnant woman wish to participate in the FAS? [Yes/No/Not asked/woman thinks about it/not applicable].

### Statistical Analysis

To describe the baseline characteristics, frequency tests were performed. Chi square tests were appliedto identify differences in the provision procedure and the intention to participate across origin, immigrant generation and Dutch language proficiency.

Associations among background characteristics, the information provision procedure of prenatal screening, the desire for information and the intention to participate in prenatal screening were examined using multivariate logistic regression. For the subanalyses of intentional participation of ethnic and language groups after adequate counseling, we used Chi square tests and multivariate logistic regression. Categorical variables related to the provision of prenatal screening and intention to participate were set as dependent variables (dependent box). Predictive variables such as age, urbanity, ethnicity, generation and language proficiency were introduced simultaneously (enter method) into the regression analyses. ‘Goodness of fit’ of the models was tested using the Hosmer–Lemeshow test. All analyses were performed using IBM SPSS statistical software version 21 (SPSS Inc., Chicago, IL, USA). For all analyses, a p-value of <0.05 was considered statistically significant.

## Results

During the study period, 31,573 registrations were collected (Table 1). Almost 5% of all cases (n = 1477) were excluded from the analysis as a result of incomplete registration, e.g., lacking a year of birth or gestational age, or lacking information on the provision and intention to participate in PS. 86% of the women were younger than 36 years old. 31% of pregnant women were first-generation immigrants. 21% had their first antenatal visit after eleven (11 + 0) weeks of gestational age. Finally, 7% had an ‘absent’ or a ‘limited’ Dutch LPL.

Table 2 shows that the first-generation immigrants were significantly more likely to be ‘too late’ for antenatal visits (first antenatal visit ≥11 + 0 weeks of gestational age) compared to the native Dutch and second-generation immigrants. Also, the results indicated that pregnant women with ‘absence of LPL’ were significantly more (41%) likely to be ‘too late’ for their first antenatal visit. The information about the CT was offered significantly less often to the first-generation immigrant group (ranging from 10 to 19% less) and to the ‘absent’ and ‘limited LPL’ groups (19 and 12% less, respectively) compared to the native Dutch, second-generation immigrant and ‘fluent LPL’ groups. Counseling about the CT and FAS was offered significantly less often to the ‘absent LPL’ group compared to the ‘fluent LPL’ group (12% less for the CT and 11% less for the FAS). The first-generation immigrants and ‘absence of LPL’ groups received the explanatory leaflet about the CT as part of the counseling process significantly less often than the other native Dutch, second-generation immigrant, and language groups (ranging from 5 to 16% less). Compared to the other LPL groups, the ‘absent LPL’ group received an explanatory leaflet about the FAS significantly less often (ranging from 10 to 12% less).

**Table 2:** Provision of prenatal screening and intentional participation in prenatal screening with CT and FAS

	Within native Dutch and immigrant generations								Within language proficiency level (LPL) group							
	Total		Native Dutch		First-gen. immigrant		Second-gen. immigrant		Total		Absent Dutch LPL		Limited Dutch LPL		Fluent Dutch LPL	
	n	(%)	n	(%)	n	(%)	n	(%)	n	(%)	n	(%)	n	(%)	n	(%)
First antenatal visit																
Late (≥11 weeks gestational age)	1175	(19)	609	15^e, f^	526	(28)^e, g^	40	(22)^e, g^	5765	(21)	235	(41)^b, c^	416	(34)^b, d^	5114	(20)^c, d^
Step 1																
Information offer																
CT (yes)	5103	(90)	3450	(94)^e^	1492	(84)^e, g^	161	(89)^g^	24,030	(89)	373	(71)^b, c^	937	(78)^b, d^	22,720	(90)^c, d^
FAS (yes)	5470	(96)	3571	(97)^e^	1723	(95)^e^	176	(98)	25,163	(94)	485	(90)^b, c^	1123	(93)^b^	23,555	(94) ^c^
Counseling desired																
CT (yes)	3960	(73)	2626	(73)	1204	(72)	130	(76)	17,996	(76)	287	(80)^b, c^	732	(87)^b^	16,977	(90)^c^
FAS (yes)	5377	(96)	3499	(96)	1707	(96)	171	(97)	23,351	(98)	465	(97)	1075	(99)	21,811	(99)
Step 2																
Counseling																
CT (yes)	4009	(76)	2668	(76)^e^	126	(77)^e^	1215	(74)	19,503	(79)	303	(67)^b, c^	789	(75)^b d^	18,411	(79)^c, d^
FAS (yes)	3956	(77)	2522	(75)^e, f^	1284	(79)^e, g^	150	(90)^f, g^	17,477	(74)	296	(64)^b, c^	755	(70)^b, d^	16,426	(75)^c, d^
Counseling setting																
Specific counseling consult	1294	(24)	829	(24)	428	(26)	37	(23)	1749	(23)	74	(37)^b, c^	104	(27)^b, d^	571	(23)^c, d^
Other	4009	(76)	2668	(76)	1,215	(74)	126	(77)	5826	(77)	126	(63)^b, c^	277	(73)^b, d^	5423	(77)^c, d^
Counseling CT^a^																
Leaflets	3495	(60)	2834	(61)^e^	1033	(56)^e, g^	122	(66)	16,089	(59)	238	(44)^b, c^	85	(56)^b, d^	5611	(60)^c, d^
Counseling (verbal explanation)	3511	(61)	2329	(62)^e^	1063	(57)^e^	119	(65)	17,441	(64)	237	(43)^b, c^	719	(56)^b, d^	16,485	(65)^c, d^
Website	945	(16)	626	(17)^e, f^	303	(16)^e, g^	16	(9)^f, g^	4673	(17)	64	(12)^b, c^	208	(17) ^b^	4401	(17) ^c^
Other	17	(<1)	7	(<1)^e^	10	(<1)^e^	0	(0)	204	(<1)	21	(4)^b, c^	19	(2)^b, d^	164	(1)^c, d^
Counseling FAS^a^																
Leaflets	4776	(83)	3072	(82)	1544	(84)	160	(87)	6753	(82)	170	(70)^b, c^	343	(80)^b^	6240	(82)^c^
Counseling (verbal explanation)	4683	(81)	3001	(80)^f^	1521	(82)	161	(87)^f^	21,956	(82)	428	(78)^c^	1003	(82)	20,525	(82)^c^
Website	988	(17)	637	(17)^f^	331	(18)^g^	20	(11)^f, g^	3952	(15)	75	(14)	192	(16)	3685	(15)
Other	110	(2)	77	(2)	32	(2)	1	(<1)	433	(5)	27	(11)^b, c^	32	(7)^b^	374	(5)^c^
Step 3																
Intention to participate in CT^a^																
Yes	1250	(22)	966	26)^e, f^	259	15)^e, g^	25	(14)^e, f, g^	6084	(23)	66	(13)^c^	124	(11)^d^	5894	(24)^c, d^
No	2524	(44)	1638	(44)^e, f^	810	(46)^e, g^	76	(42)^e, f, g^	11,733	(44)	199	(40)^c^	551	(47)^d^	10,983	(44)^c, d^
Thinking about	1338	(23)	849	(23)^e, f^	429	(25)^e, g^	60	(33)^e, f, g^	6310	(24)	102	(21)^c^	270	(23)^d^	5938	(24)^c, d^
Inapplicable/not asked	581	(10)	255	(7)^e, f^	255	(14)^e, g^	21	(11)^e, f, g^	2526	(9)	127	(26)^c^	227	(19)^d^	1879	(8)^c, d^
Intention to participate in FAS^a^																
Yes	5009	(88)	3286	(89)	1562	(88)	161	(88)	22,312	(84)	439	(84) ^c^	993	(85)	20,880	(85) ^c^
No	49	(<1)	31	(<1)	17	(1)	1	(<1)	443	(2)	10	(2) ^c^	19	(2)	414	(2) ^c^
Thinking about	412	(7)	261	(7)	137	(8)	14	(8)	2423	(9)	38	(7)^c^	103	(9)	2282	(9)^c^
Inapplicable/not asked	220	(4)	128	(3)	86	(5)	6	(3)	1470	(5)	33	(6)^c^	60	(5)	1046	(4)^c^

Significant differences = p-value between 0.001 and 0.05

*PS* prenatal screening, *CT* combined test, *FAS* fetal anomaly scan, *gen* generation

^a^More than one answer is possible

^b^Significant difference between *absent* Dutch language proficiency level and *limited* Dutch language proficiency level

^c^Significant difference between *absent* Dutch language proficiency level and *fluent* Dutch language proficiency level

^d^ Significant difference between *limited* Dutch language proficiency level and *fluent* Dutch language proficiency level

^e^Significant difference between native Dutch and first-generation immigrants

^f^Significant difference between native Dutch and second-generation immigrants

^g^Significant difference between first- and second-generation immigrants

Table 3 shows that pregnant women with an ‘absence of LPL’ were less likely to get an information offer about the CT (OR 0.40; 95% CI 0.23–0.71) and to be given counseling about the CT (OR 0.48; 95% C: 0.38–0.68). A non-urban or less urban maternal residency was associated with a lower chance that information about the FAS was offered (O, 0.65; 95% C: 0.45–0.96) and that counseling was received about the CT (moderate urban: OR 0.64; 95% C: 0.45–0.75 and non-urban/less urban: OR 0.79; 95% CI 0.64–0.97). Pregnant women with an absence of Dutch LPL were associated with a lower desire for counseling about prenatal screening for both the CT (OR 0.45; 95% CI 0.31–0.63) and the FAS (OR 0.45; 95% CI 0.23–0.91). There was an association found between pregnant women living in non-urban or less urban areas and a reduced wish for counseling about the FAS (OR 0.39; 95% CI 0.27–0.58). Compared to the native Dutch group, a substantially increased desire for counseling on the CT was found for the Cape Verdean ethnic group (OR 2.48; 95% CI 1.09–5.68). Pregnant women with a first-generation immigrant background were associated with a higher chance of receiving counseling about the FAS (OR 1.91; 95% CI 1.11–3.30). Living in non-urban or less urban areas was associated with lower intention to take up PS with the CT (OR 0.55; 95% CI 0.44–0.68). When a pregnant woman had Surinamese, Dutch Antillean, Turkish or Moroccan ethnicity, there was a significant negative association with intention to participate in the CT. An ‘absent’ and ‘limited LPL’ status had a significantly negative association with intention to participate in the CT (range OR 0.46–0.48) and the FAS (range OR 0.45–0.49).

**Table 3:** Multivariate association between background characteristics and provision and intentional participation in prenatal screening with CT and FAS

	Step 1								Step 2				Step 3			
	Information offer CT		Counseling desired CT		Information offer FAS		Counseling desired FAS		Counseling CT		Counseling FAS		Intention to participate in CT		Intention to participate in FAS	
	n = 5386		n = 5189		n = 5429		n = 5312		n = 5056		n = 4927		n = 5425		n = 5422	
	OR	CI (95%)	OR	CI (95%)	OR	CI (95%)	OR	CI (95%)	OR	CI (95%)	OR	CI (95%)	OR	CI (95%)	OR	CI (95%)
Age	*		*						*		*		*		*	
30–39	Ref		Ref		Ref		Ref		Ref		Ref		Ref		Ref	
≤19	**0.41**	**(0.24–0.70)** ^**§**^	**0.96**	(0.59–1.57)	0.73	(0.29–1.85)	0.85	(0.30–2.40)	0.85	(0.51–1.39)	**0.96**	(0.57–1.63)	**0.21**	**(0.10–0.45)** ^**§**^	**0.37**	**(0.22–0.61)** ^**§**^
20–29	**0.87**	(0.71–1.05)	**0.82**	**(0.72–0.93)** ^**‡**^	1.00	(0.76–1.34)	1.21	(0.90–1.64)	0.85	**(0.75–0.97)** ^**†**^	**0.73**	**(0.64–0.85)** ^**§**^	**0.33**	**(0.29–0.39)** ^**a**^	**0.76**	**(0.64–0.91)** ^**‡**^
40–50	**0.36**	**(0.22–0.57)** ^**§**^	**0.91**	(0.59–1.40)	0.45	**(0.23–0.91)** ^**†**^	0.75	(0.32–1.76)	0.58	**(0.39–0.86)** ^**‡**^	**1.32**	(0.86–2.03)	**2.18**	**(1.49–3.19)** ^**§**^	**0.72**	(0.42–1.22)
Urbanity			*				*		*		*		*		*	
Highly urban	Ref		Ref				Ref		Ref		Ref		Ref		**Ref**	
Moderately urban	1.05	(0.82–1.36)	**0.75**	**(0.64–0.87)** ^**§**^	0.90	(0.63–1.30)	**0.65**	**(0.45–0.95)** ^**†**^	**0.64**	**(0.45–0.75)** ^**§**^	**1.05**	(0.90–0.23)	**0.68**	**(0.58–0.80)** ^**§**^	**0.56**	**(0.56–0.68)** ^**§**^
Not or less urban	1.08	(0.81–1.46)	**0.62**	**(0.52–0.75)** ^**§**^	0.65	**(0.45–0.96)** ^**†**^	**0.39**	**(0.27–0.58)** ^**§**^	**0.79**	**(0.64–0.97)** ^**†**^	**0.06**	(0.04–0.10)	**0.55**	**(0.44–0.68)** ^**§**^	**0.90**	(0.69–1.16)
Ethnicity			*						*		*		*			
Dutch	Ref		**Ref**		Ref		Ref		Ref		Ref		**Ref**		Ref	
Surinamese	0.61	(0.32–1.18)	**1.42**	(0.85–2.36)	1.11	(0.36–3.48)	1.84	(0.44–7.69)	1.22	(0.72–2.09)	**0.31**	**(0.16–0.60)** ^**§**^	**0.40**	**(0.21–0.75)** ^**‡**^	0.81	(0.43–1.54)
Antillean	0.56	(0.28–1.14)	**0.94**	(0.55–1.60)	1.07	(0.31–3.66)	0.85	(0.23–3.08)	0.66	(0.38–1.14)	**0.44**	**(0.22–0.87)** ^**†**^	**0.25**	**(0.11–0.55)** ^**§**^	1.64	(0.71–3.76)
Cape Verdean	0.78	(0.31–1.98)	**2.48**	**(1.09–5.68)** ^**†**^	1.20	(0.24–6.06)	n.a	n.a	1.82	(0.79–4.18)	**0.29**	**(0.12–0.70)** ^**‡**^	**0.7** **5**	(0.35–1.63)	1.26	(0.45–3.49)
Turkish	0.74	(0.39–1.35)	**1.09**	(0.70–1.71)	1.05	(0.39–2.85)	1.24	(0.40–3.82)	1.05	(0.66–1.68)	**0.47**	**(0.25–0.85)** ^**†**^	**0.39**	**(0.22–0.71)** ^**‡**^	0.85	(0.47–1.51)
Moroccan	0.60	(0.34–1.05)	**0.83**	(0.55–1.26)	1.08	(0.43–2.76)	0.89	(0.33–2.42)	0.81	(0.53–1.26)	**0.30**	**(0.16–0.54)** ^**§**^	**0.14**	**(0.07–0.27)** ^**§**^	0.94	(0.54–1.63)
Other	0.67	(0.38–1.16)	**1.59**	**(1.05–2.40)** ^**†**^	0.89	(0.38–2.17)	1.20	(0.45–3.19)	1.11	(0.73–1.70)	**0.49**	**(0.28–0.85)** ^**†**^	**0.90**	(0.55–1.47)	0.91	(0.54–1.54)
Generation^e^											*					
Native Dutch	Ref		Ref		Ref		Ref		Ref		**Ref**		Ref		Ref	
First	0.74	(0.40–1.35)	0.84	(0.57–1.24)	0.81	(0.34–1.92)	0.85	(0.33–2.21)	0.90	(0.60–1.36)	**1.91**	**(1.11–3.30)** ^**†**^	1.04	(0.63–1.70)	1.04	(0.63–1.72)
Language proficiency level (LPL)			*		*				*				*		*	
Fluent	Ref		Ref		Ref		Ref		Ref				Ref		Ref	
Limited	0.55	(0.37–0.82)^**‡**^	**0.76**	(0.57–1.01)	**0.61**	(0.36–1.03)	0.98	(0.49–1.95)	0.75	**(0.56–1.00)** ^**†**^	0.90	(0.65–1.24)	**0.46**	**(0.31–0.68)** ^**§**^	**0.49**	**(0.35–0.69)** ^**§**^
Absent	0.38	(0.23–0.61)^§^	**0.45**	**(0.31–0.63)** ^**§**^	**0.40**	**(0.23–0.71)** ^**†**^	**0.45** ^**†**^	**(0.23–0.91)**	**0.48**	**(0.38–0.68)** ^**§**^	0.74	(0.48–1.15)	**0.48**	**(0.30–0.77)** ^**‡**^	**0.45**	**(0.30–0.37)** ^**§**^

Adjusted for all predictor variables in the table

*CT* combined test, *FAS* fetal anomaly scan

Bold is significant: ^*^Significant difference p < 0.05 within predictor variable; ^†^p < 0.05; ^‡^p < 0.01; ^§^p < 0.001

Results of second-generation immigrants were not applicable

Table 4 shows that having a ‘limited and absent LPL’ was significantly associated with a lower chance of receiving an information offer about the CT (−14%, OR 0.44 (0.33–0.58 p < 0.001) compared to the ‘fluent’ language group. If the information provision procedure about the CT is executed adequately, the intentional participation in the CT is significantly lower within the non-Western immigrant and the ‘limited and absent LPL’ groups compared to the native and Western groups and ‘fluent LPL’ group (21 and 19% less, respectively). After an adequate information offer about the FAS to pregnant women with a non-Western immigrant and a ‘limited and absent LPL’ background, these groups showed significantly lower percentages of counseling about the FAS in comparison with the native and Western groups and ‘fluent LPL’ group (5 and 6%, respectively).

**Table 4:** Intentional participation of ethnicity and language groups after adequate information provision prenatal screening with CT and FAS

	Step 1					Step 2	Step 3	
	Total	Information offer CT		Counseling desired CT		Counseling CT	Intention to participate in CT	
		Yes n (%)^g^	OR CI (95%)^h^	Yes n (%)^a, g^	OR CI (95%)^h^	Yes n (%)^b, g^	Yes n (%)^c, g^	OR CI (95%)^h^
Ethnicity^d, e^								
Native/Western	3877	**3606 (93)** ^**‡**^	Ref	**2761 (77)** ^**†**^	Ref	2653 (96)	**879 (32)** ^**‡**^	Ref
Non-Western	889	**756 (85)**	1.05 (0.65–1.71)	**571 (76)** ^†^	0.70 (0.46–1.05)	538 (94)	**64 (11)** ^**‡**^	1.61 (0.84–3.10)
Language^f^								
Fluent	25,244	**22,720 (90)** ^**‡**^	**Ref**	**16,997 (75)** ^†^	**Ref**	16,638 (98)	**4977 (46)** ^**‡**^	Ref
Limited/absent	1723	**1310 (76)** ^**‡**^	**0.44 (0.33–0.58)** ^**§**^	**1019 (78)** ^**†**^	**0.62 (0.49–0.79)** ^**‡**^	988 (97)	**167 (27)** ^**‡**^	1.93 (1.29–2.88)

	Step 1					Step 2	Step 3	
	Total	Information offer FAS		Counseling desired FAS		Counseling FAS	Intention to participate in FAS	
		Yes n (%)^g^	OR CI (95%)^h^	Yes n (%)^a, g^	OR CI (95%)^h^	Yes n (%)^b, g^	Yes n (%)^c, g^	OR CI (95%)^h^
Ethnicity^d, h^								
Native/Western	3865	**3749 (97)** ^**‡**^	Ref	3674 (98)	Ref	**863 (23)** ^**†**^	813 (94)	
Non-Western	902	**866 (96)** ^**‡**^	1 (0.41–2.44)	857 (99)	0.71 (0.42–1.22)	**158 (18)** ^**†**^	141 (89)	n.a
Language^f^								
Fluent	25,058	**23,555 (94)** ^**‡**^	**Ref**	21,811 (93)	Ref	**15,766 (72)** ^**‡**^	14,015 (89)	Ref
Limited/absent	1748	**1608 (92)** ^**‡**^	**1.92 (1.24–2.99)** ^†^	1540 (96)	1.41 (0.97–2.04)	**1,021 (66)** ^**‡**^	921 (90)	2.26 (0.17–30)

*CT* combined test, *FAS* fetal anomaly scan, *n.a*. not applicable

Bold is significant: ^†^p < 0.01, ^‡^p < 0.001

^a^Percentage based on the number of pregnant women that had an information offer about CT/FAS

^b^Percentage based on the number of pregnant women that desired counseling about CT/FAS

^c^Percentage based on number of pregnant women that was counseled about CT/FAS

^d^Adjusted for age, gravidity, parity, immigrant generation, urbanity and language proficiency

^e^The ‘other’ ethnicity category, presented in different analyses in this article, is excluded from analyses due to uncertainty about Western and non-Western backgrounds because the country of birth of the parents of pregnant women is missing

^f^Adjusted for age, gravidity, parity, immigrant generation, urbanity and ethnicity

^g^Chi^2^ testing

^h^Logistic regression analyses

## Discussion

The results of this study indicate that there are disparities in the provision of prenatal screening (PS) across ethnic and language proficiency subpopulations. These disparities can possibly result in insufficient access to prenatal screening. This insufficient access may be explained by (1) cultural barriers, (2) language barriers, (3) delays in attending the first antenatal visit, (4) unfamiliarity with diversity by practitioners, and (5) the attitude of the counselor. The literature suggests that the growth of the number of patients born abroad with a variety of social, cultural and religious affiliations and migration histories and statuses influences healthcare provision and reception (Andrulis 1998; Nkulu Kalengayi et al. 2012; Gitsels-van der Wal et al. 2014b; Harmsen et al. 2008). This growth could explain the lack of the provision of and wish for counseling about the CT and the FAS among non-Western women in this study. Several studies show that language barriers are a threat to the effective provision and actual use of health services, which is in line with the positive association between the demand for counseling for the CT and the FAS and Dutch language proficiency found in this study (Thomas et al. 2010; Nkulu Kalengayi 2012; Schouten and Meeuwesen 2006; Santibañez et al. 2015). Immigrant women’s underutilization of midwifery services may be linked to the delay in the first antenatal visit (Otero-Garcia et al. 2013). Along with other studies, our study shows a higher percentage of first-generation immigrant women and women with an absence of limited language proficiency who are late for their first antenatal visit (Alderliesten et al. 2007). A late start of antenatal care could be a reason for less frequent offers of information about PS and a lower provision of counseling about PS, especially about the first trimester CT. This lower provision of counseling can decrease the chance for accurate participation in prenatal screening. Additionally, specific behaviors and attitudes by healthcare providers towards immigrants cause mutual mistrust. Practitioners fear that they might unintentionally discriminate against immigrant patients because they think they are not knowledgeable and/or have no skills to address the varied social, political and cultural backgrounds of these patients. This apprehension might be responsible for the deficiency in the information provision procedure for prenatal screening of non-Western pregnant women in our study (Thomas et al. 2010). An interesting outcome of this study is the significant association between having a Cape Verdean ethnicity and an increased wish for counseling on the CT. A possible explanation for this finding is the general negative attitude towards the phenomenon of ‘disability’ within the Cape Verdean culture (Thomas DM, 2009). Another explanation could be the fact that in comparison to other non-Western Dutch pregnant women, a high percentage of the Dutch Cape Verdean community lives in highly urbanized areas (Statistics Netherlands 2013, 2004). In highly urbanized areas, an increased demand for counseling about the CT is generally observed. In addition, since the total percentage of Cape Verdean women in the study is very small, this finding might be coincidental.

The legislative framework of the Dutch prenatal screening program requires ‘verbal explanation’ and (translated) ‘leaflets’ about prenatal screening as a minimum necessary element of pretest counseling. An interpreter is not available free of charge. In this study, pregnant women with an ‘absent or limited LPL’ received an informational prenatal screening leaflet about the CT or FAS less often, which indicates that part of the population of pregnant women is being underserved in terms of receiving written information about prenatal screening (National Institute for Public Health and Environment et al. 2007). This finding can be explained by (1) a potentially negative attitude among practitioners towards the use of leaflets based on previous outcomes regarding the ineffectiveness of leaflets in a medical setting (Gal and Prigat 2005; Kloza et al. 2014), (2) a lack of knowledge of the counselors about the availability of multilingual leaflets, and (3) a possible lack of motivation or time to give these leaflets to patients (multilingual leaflets must be downloaded and printed) (Bungartz et al. 2011). These assumptions can be confirmed by the actual use of translated leaflets about prenatal screening within the Netherlands during 2014 (see Online Appendix 2).

An association between a relatively low rate of intention to participate in the CT among Dutch immigrant women and women with an absent or limited Dutch LPL was partially confirmed in previous papers (Fransen et al. 2009, 2010b; Bungartz et al. 2011). Most Dutch pregnant women with an absent or limited LPL usually have an immigrant background (Statistics Netherlands 2008); therefore, we will only discuss outcomes for pregnant immigrant women as they relate to intention to participate. Low intention to participate among immigrant women can be explained by (1) socio-economic background, (2) age of immigrant women, (3) low awareness of prenatal screening with the CT and (4) more acceptance of a child with Down’s syndrome within the non-Western pregnant women’s population. Since Dutch citizens of Antillean, Surinamese, Turkish and Moroccan descent generally have lower incomes, they possibly experience a resistance to participate in the CT because of the obligation to pay for PS for women under the age of 36 years (Fransen et al. 2010b; Statistics Netherlands 2014c). An average income or a lack of income provides an explanation for the lower uptake for the CT (Gitsels-van der Wal et al. 2014a). In our study and in general, the population of pregnant women with these ethnic backgrounds was predominantly younger than 36 years old compared to the native Dutch population (Statistics Netherlands 2013). Earlier studies reported that Turkish pregnant women read written information less often, had little knowledge about Down’s syndrome and prenatal screening and did not make well-informed decisions as often (Fransen et al. 2010a, 2009). Several studies have reported that when pregnant women with a non-Western background do not participate in prenatal screening, a more positive opinion about birth defects based on religious beliefs play a role (Fransen et al. 2009; Gitzels-vand der Wal 2014c; Crombag et al. 2015). In this study, an association was found between intention to participate and ‘absent’ and ‘limited’ Dutch LPL groups in the FAS, such that these groups showed less frequent participation. This finding contrasts with previous data on FAS uptake, which indicates almost similar uptake rates between Dutch and non-Dutch pregnant women (Gitsels-van der Wal et al. 2014a).

### Strengths and Limitations

Comparing the demographic data of our study population to those from the general population shows an almost equal distribution in terms of age and ethnicity, which highlights the representativeness of the study (see Table 1) (Statistics Netherlands 2013). The strengths of the study are the exploration of outcomes concerning ethnicity and language proficiency differences in the provision and (intentional) participation for both the CT and the FAS, which has not been frequently studied before. The risk of confounding effects on study outcomes is less likely because the data were registered during a period in which the Dutch prenatal screening program was already implemented for one and a half years. The program was not subject to changes and there were no major socio-demographic trends in the southwestern region of the Netherlands. This study has several limitations. First, LPL is classified subjectively. Differences in interpretation may well exist between the approximately 200 prenatal healthcare providers included in our dataset. Second, as a result of the strict definition of a second-generation immigrant, data from pregnant women with this background were underrepresented in this study (Statistics Netherlands 2013). Third, a significant limitation of the study is that the data consist of intention to participate in prenatal screening and not actual participation. There may be a difference between hypothetical and actual uptake within the study population, but rates of intention to take up the CT and FAS in this study show similarities with actual CT and FAS uptake rates in the Netherlands, which is promising for the representativeness of the study results (Crombag et al. 2014; Gitsels-van der Wal et al. 2014a).

### Generalizability

The Population Screening Act for prenatal screening calls for equal access to the prenatal screening program. Therefore, the findings of this research are important for healthcare providers, policy makers and governmental professionals involved in the Dutch prenatal screening program. The study results support the development of interventions aiding in the provision of transcultural healthcare that are directed to professional counselors settled in highly urbanized areas, which are strongly multi-ethnic. The results of this study can teach health professionals outside the Netherlands more about the influence of ethnicity and language proficiency differences in the PS provision procedures and intention to participate in screening. Furthermore, culturally and socio-economically competent visual information materials, such as educational films that are sensitive to differences in culture and language proficiency, should be developed. Healthcare professionals should be encouraged to find better ways to inform their patients. Additional qualitative research among health professionals is desirable to explore why non-native Dutch women and women with limited LPL are less likely to be offered counseling on the CT and FAS. Future research should determine LPL based on several parameters: indication of LPL by both professionals and pregnant women and an optional language skill test.

## Conclusions

This study shows that pregnant women with a non-native Dutch background and/or with insufficient Dutch language proficiency are underserved more often within the Dutch prenatal screening program. These women are less likely to receive an information offer about the CT and FAS and to receive counseling about prenatal screening. In addition, these women exhibit a profoundly lower rate of intention to participate in the CT and FAS. The Population Screening Act ‘prenatal screening’ calls for equal access to the prenatal screening program. Therefore, these study findings are important for healthcare practitioners, policy makers and governmental professionals involved in the Dutch prenatal screening program.

## Electronic supplementary material

Below is the link to the electronic supplementary material.

Supplementary material 1 (DOCX 15 KB)

Supplementary material 2 (DOCX 16 KB)

Supplementary material 3 (VSD 58 KB)

## References

[CR1] Alderliesten ME, Vrijkotte TGM, van der Wal MF (2007). Late start of antenatal care among ethnic minorities in a large cohort of pregnant women. BJOG: An International Journal of Obstetrics and Gynaecology.

[CR2] Andrulis DP (1998). Access of care is the centerpiece in the elimatination of socio-economic disparities in health. Annals of Internal Medicine.

[CR3] Bakker M, Birnie E, Pajkrt E (2012). Low uptake of the combined test in the Netherlands: Which factors conribute?. Prenatal Diagnosis.

[CR4] Boyd PA, DeVigan C, Khoshnood B (2008). Survey of prenatal screening policies in Europe for structural malformations and chromosome anomalies, and their impact on detection and termination rates for neural tube defects and Down’s syndrome. BJOG.

[CR5] Bricker L, Garcia J, Henderson J (2000). Ultrasound screening in pregnancy: A systematic review of the clinical effectiveness, cost-effectiveness and women’s views. Health Technology Assess.

[CR6] Bungartz J, Szecsenyi J, Joos S (2011). He that knows nothing doubts nothing: Availability of foreign language patient education material for immigrant patients in Germany—a survey]. Zeitschrift für Evidenz, Fortbildung und Qualität im Gesundheitswesen.

[CR7] Crombag NM, Bensing JM, Iedema-Kuiper R (2013). Determinants affecting pregnant women’s utilization of prenatal screening for Down syndrome: A review of the literature. The Journal of Maternal-Fetal & Neonatal Medicine.

[CR8] Crombag NM, Vellinga YE, Kluijfhout SA (2014). Explaining variation in Down’s syndrome screening uptake: comparing the Netherlands with England and Denmark using documentary analysis and expert stakeholder interviews. BMC Health Services Research.

[CR9] Crombag NMTH, Schielen PCJI, Hukkelhoven CW (2015). Determinants of first trimester combined test participation within the central region of the Netherlands. Prenatal Diagnosis.

[CR10] Dormandy E, Hooper R, Michie S (2002). Informed choice to undergo prenatal screening: A comparison of two hospitals conducting testing either as part of a routine visit or requiring a separate visit. Journal of medical screening.

[CR11] Dormandy E, Michie S, Hooper R (2005). Low uptake of prenatal screening for Down syndrome in minority ethnic groups and socially deprived groups: A reflection of women’s attitudes or a failure to facilitate informed choices?. International Journal of Epidemiology.

[CR12] Fransen MP, Essink-Bot M-L, Vogel I (2010). Ethnic differences in informed decision-making about prenatal screening for Down’s syndrome. Journal of Epidemiology & Community Health.

[CR13] Fransen MP, Schoonen HMHJD, Mackenbach JP (2010). Ethnic differences in participation in prenatal screening for Down syndrome: A register-based study. Prenatal Diagnosis.

[CR14] Fransen MP, Wildschut HIJ, Vogel I (2009). Ethnic differences in considerations whether or not to participate in prenatal screening for Down syndrome. Prenatal Diagnosis.

[CR15] Gal I, Prigat A (2005). Why organizations continue to create patient information leaflets with readability and usability problems: An exploratory study. Health Education Research.

[CR16] Gitsels-van der Wal JT, Verhoeven PS, Manniën J (2014). Factors affecting the uptake of prenatal screening tests for congenital anomalies; a multicentre prospective cohort study. BMC Pregnancy and Childbirth.

[CR17] Gitzels-van der Wal JT, Manniën J, Gitzels LA (2014). Prenatal screening for congenital anomalies exploring midwives’ perceptions of counseling clients with religious backgrounds. BMC Pregnancy and Childbirth.

[CR18] Gitzels-van der Wal JT, Manniën J, Mohammed MG (2014). The role of religion in decision-making on antenatal screening of congenital anomalies: A qualitative study amongst Muslim Turkish origing immigrants. Midwifery.

[CR19] Harmsen JA, Bernsen RMD, Bruijnzeels MA (2008). Patients’ evaluation of quality of care in general practice: what are the cultural and linguistic barriers?. Patient Education and counseling.

[CR20] Health Council of the Netherlands. (2007). Population screening act: Prenatal Screening for Down’s syndrome and neural tube defects. Retrieved November 10, 2014, from http://www.gr.nl/sites/default/files/200705WBO.pdf.

[CR21] Kloza EM, Haddow PK, Halliday JV (2014). Journal of Genetic Counseling.

[CR22] Marteau TM, Dormandy E, Michie SA (2001). Measure of informed choice. Health Expect.

[CR23] National Institute for Public Health and Environment. (2011). In H. M. E. Van Agt, H. M. H. J. D. Schoonen & J. Fracheboud, H. J. de Koning (Eds.). National and regional monitor Informed Decision Making Prenatal Screening. Bilthoven. Retrieved May 20, 2016, from http://www.rivm.nl/dsresource?objectid=7bea4619-78b3-4b93-8483 d515b89dd710&type = org&disposition = inline.

[CR42] National Institute for Public Health and Environment., Waelput, A. J. M., & Achterberg, P. W. (2007). Ethnic origin and care during pregnancy and birth: An exploration of Dutch research, Bilthoven. Retrieved October 15, 2015, from http://www.rivm.nl/en/Documents_and_publications/Scientific/Reports/2008/maart/Ethnic_origin_and_care_during_pregnancy_and_birth_an_exploration_of_Dutch_research.

[CR24] Nivel. (2015). Informed/uninformed decision making for uptake prenatal screening for Down’s syndrome and congenital anomalies of pregnant women of Turkish and Maroccan background, pregnant women with a low SES and young pregnant women. Retrieved April 18, 2015, from http://www.nivel.nl/sites/default/files/bestanden/Rapport-prenatale-screening-downsyndroom.pdf.

[CR25] Nkulu Kalengayi FK, Hurtig A-K, Ahlm C (2012). “It is a challenge to do it the right way”: an interpretive description of caregivers’ experiences in caring for migrant patients in Northern Sweden. BMC Health Services Research.

[CR26] Otero-Garcia L, Goicolea I, Gea-Sánchez M (2013). Access to and use of sexual and reproductive health services provided by midwives among rural immigrant women in Spain: Midwives’ perspectives. Global Health Action.

[CR27] Perinatal Registration the Netherlands. (2014). Dataset Perinatal Registration 1b. Retrieved October 20, 2015, from http://www.perinatreg.nl/wat_wordt_geregistreerd.

[CR28] Santibáñez M, Paz-Zulueta M, Ruiz M (2015). Factors associated with lack of adherence to antenatal care in African immigrant women and Spanish women in northern Spain: The role of social risk factors in combination with language proficiency. Midwifery.

[CR29] Schouten BC, Meeuwesen L (2006). Cultural differences in medical communication: A review of the literature. Patient Education and Counseling.

[CR30] Slaughter-Acey JC, Caldwell CH, Misra DP (2013). The influence of personal and group racism on entry into prenatal care among African American women. Women’s Health Issues: Official Publication of the Jacobs Institute of Women’s Health.

[CR31] Statistics Netherlands. (2004). Cape Verdeans in the Netherlands. Retrieved January 7, 2015, from http://www.cbs.nl/NR/rdonlyres/06F53672-29A8-4BC7-913D-55B916FA2A3B/0/2004k3b15p085art.pdf.

[CR32] Statistics Netherlands. (2008). Language proficiency Maroccan, Turkish, Antillian, Surinamese immigrants in the Netherlands. Retrieved December 12, 2014, from http://www.cbs.nl/nl-NL/menu/themas/dossiers/allochtonen/publicaties/artikelen/archief/2008/2008-2570-wm.html.

[CR33] Statistics Netherlands. (2013). Data request: Exact numbers mother who gave birth to living child Southwest region 2007–2012 [postal code, ethinicity, immigrant generation and age].

[CR34] Statistics Netherlands. (2014a). Standard definition immigrant. Retrieved November 15, 2014, from http://www.cbs.nl/NR/rdonlyres/26785779-AAFE-4B39-AD07-59F34DCD44C8/0/index1119.pdf.

[CR35] Statistics Netherlands. (2014b). Urbanity of a area. Retrieved September 10, 2014, from http://www.cbs.nl/nl-NL/menu/methoden/begrippen/default.htm?ConceptID=658.

[CR43] Statistics Netherlands. (2014c). Avarage income, to characteristics households. Retrieved January 17, 2015, from http://statline.cbs.nl/StatWeb/publication/?VW=T&DM=SLNL&PA=70843ned&D1=0,6&D2=0-1,9,15,18&D3=17-18,23-28,34-35&D4=l&HD=091217-1446&HDR=G3,T,G1&STB=G2.

[CR36] Statistics Netherlands. (2015). Concepts ethnic background [Dutch: begrippen herkomstgroepering]. Retrieved January 10, 2015, from http://www.cbs.nl/nl-NL/menu/methoden/begrippen/default.htm?ConceptID=315.

[CR37] Thomas DM (2009). Culture and disability: A Cape Verdean perspective. Journal of Cultural Diversity.

[CR38] Thomas PE, Beckmann M, Gibbons K (2010). The effect of cultural and linguistic diversity on pregnancy outcome. The Australian & New Zealand Journal of Obstetrics & Gynaecology.

[CR39] Vassy C, Rosman S, Rousseau B (2014). From Policy making to service use. Down’s syndrome antenatal screening in England, France and the Netherlands. Social Science & Medicine.

[CR40] Wald NJ, Hackshaw AK (1997). Combining ultrasound and biochemistry in first-trimester screening for Down’s syndrome. Prenatal Diagnosis.

[CR41] Woloshin S, Schwartz LM, Katz SJ (1997). Is language a barrier to the use of preventive services?. Journal of General Internal Medicine.

