# Supplementary material for: Ethnicity and Language Proficiency Differences in the Provision of and Intention to Use Prenatal Screening for Down’s Syndrome and Congenital Anomalies. A Prospective, Non-selected, Register-Based Study in the Netherlands
Source: Matern Child Health J. 2017 Sep 7;22(3):343–54. doi: 10.1007/s10995-017-2364-2 (PMC5845051; doi:10.1007/s10995-017-2364-2)
Supplement: Supplementary file 1 — Supplementary material 1 (DOCX 15 KB) [file 10995_2017_2364_MOESM1_ESM.docx]

| **Appendix I.** Registration of women’s socio-demographic and pregnancy characteristics in the web-based form | | |
| --- | --- | --- |
| ***Variable*** | ***Defined on*** | ***Answer options / categories*** |
| *Age* | year of birth |  |
| *Ethnic background* | Country of birth of the woman and her parents | *First generation:* ethnic background determined by the country of birth of the pregnant woman.  *Second generation:* ethnic background determined by the country of birth of her mother. If her mother is born in the Netherlands, then the country of birth of her father is leading  (Statistics Netherlands, 2015). |
| *Migrant generation* | Country of birth of the woman and her parents | *First-generation* immigrants are born abroad; *second-generation* immigrants are born in the Netherlands and have at least one parent born abroad (Statistics Netherlands, 2014a). |
| *Parity* | Number of live births | Nulliparous, multiparous 1-3 or 4-14, gravidity |
| *Gravity* | Number of pregnancies | 0-2 or >2 pregnancies |
| *Gestational age* | Gestational age during first booking visit. Weeks of gestation dated by last menstrual period (LMP) |  |
| *First booking visit* | Gestational age during first booking visit | *On time:* 0-11 weeks gestational age (counseling for combined test possible)  *Too late:* >11 (11+0) weeks gestational age |
| *Dutch language proficiency level (LPL)* | Determined and registered by a prenatal healthcare professional | *‘Fluent’*: woman speaks Dutch sufficiently, or *‘Limited’*: woman speaks Dutch with difficulty or *‘Absent’*: woman does not speak Dutch and/or communication is impossible without a translator (Perinatal Registration the Netherlands, 2014). |
| *Urbanity* | The four digit zip code of the healthcare organization was used to determine the urbanity category of the pregnant women. | *Not or less urban:* mean area address density (aad) <500-1000 addresses per square km, *moderately urban:* aad 1000 tot < 1500 addresses per km^2^ and *highly urban:* aad > 1500- >2500 addresses per square km (Statistics Netherlands, 2014^b^). |
