# Supplementary material for: Ethnicity and Language Proficiency Differences in the Provision of and Intention to Use Prenatal Screening for Down’s Syndrome and Congenital Anomalies. A Prospective, Non-selected, Register-Based Study in the Netherlands
Source: Matern Child Health J. 2017 Sep 7;22(3):343–54. doi: 10.1007/s10995-017-2364-2 (PMC5845051; doi:10.1007/s10995-017-2364-2)
Supplement: Supplementary file 2 — Supplementary material 2 (DOCX 16 KB) [file 10995_2017_2364_MOESM2_ESM.docx]

| **Appendix II. Use of translated hardcopy and downloaded leaflets about Dutch prenatal screening in 2014** | | | | | | | |
| --- | --- | --- | --- | --- | --- | --- | --- |
| **Language** | **Birth rates *** | **First immigrant generation *†** | **Difficulty with reading Dutch §** | **Use of leaflet CT ^¶^** | **Possibly no translated leaflets CT provided** | **Use of leaflets FAS ^¶^** | **Possibly no translated leaflet FAS provided** |
|  | n= (%) | n= (%) | n= (%) | n= (%) | n= (%) | n= (%) | n= (%) |
| ***Dutch*** | 126.259 †(74) |  |  | 170.542 **(98) |  | 170.138 ** (99) |  |
| ***Total Western immigrants*** | 17.635 (10) | 10.406 (59)* |  |  |  |  |  |
| *English* |  |  |  | 1.090 (0,6) |  | 593 (0,3) |  |
| *Spanish* |  |  |  | 208 (0,1) |  | 128 (0,1) |  |
| *French* |  |  |  | 149 (0,1) |  | 80 (0) |  |
| *German* |  |  |  | 141 (0,1) |  | 76 (0) |  |
| ***Total non-Western immigrants*** | 27.447 (16) | 18.961 (69)†† |  |  |  |  |  |
| *Turkish* | 5.873 (3)†† | 2.978 (51)†† | 983 (33)†† | 345 (0,2) | 638 (65) ‡‡ | 168 (0,1) | 815 (83) ‡‡ |
| *Arabic (Moroccan)* | 7.424 (4)†† | 4.432 (60)†† | 1.063 (24)†† | 320 (0,2) | 743 (70) ‡‡ | 257 (0,1) | 806 (76) ‡‡ |
| *Chinese* |  |  |  | 268 (0,2) |  | 167 (0,1) |  |
| *Portuguese (Cape Verdean)* |  |  |  | 134 (0,1) |  | 65 (0) |  |
| *Papiamento (Antillean)* | 2.199 (1)†† | 1.371 (62)†† | 41 (3)†† | 109 (0,1) | adequate | 45 (0) | adequate |
| **Total** | **171.341** |  |  | **173.306** |  | **171.717** |  |
| ** Statistics Netherlands Statline: birth rates by ethnicity until December 31, 2013. Ref: http://statline.cbs.nl/Statweb/publication/?DM=SLNL&PA=37884&D1=a&D2=0&D3=0-2,4-9&D4=14-17&HDR=T&STB=G2,G1,G3&VW=T*  *† Dutch and Surinamese; both Dutch speaking*  *‡ More need for translated leaflets within first-generation immigrant groups*  *§ Statistics Netherlands; difficulty with reading in Dutch, first generation 2006 Ref: http://www.cbs.nl/nl-NL/menu/themas/dossiers/allochtonen/publicaties/artikelen/archief/2008/2008-2570-wm.htm*  *¶ Data request: National Institute for Public Health & Environment (2014).*  *** Inclusive 170.000 Dutch hardcopy leaflets for both CT and FAS*  *†† Percentage within ethnicity / language group*  *‡‡ Percentage: the number of pregnant women who have difficulty reading in Dutch* ***minus*** *the* *number of downloads translated leaflet CT/FAS gave the number and percentage of absence* | | | | | | | |
